# Supplementary material for: dCas9-Based PDGFR–β Activation ADSCs Accelerate Wound Healing in Diabetic Mice through Angiogenesis and ECM Remodeling
Source: Int J Mol Sci. 2023 Mar 21;24(6):5949. doi: 10.3390/ijms24065949 (PMC10057415; doi:10.3390/ijms24065949)
Supplement: Supplementary file 1 [file ijms-24-05949-s001.zip › Supplementary files.pdf]

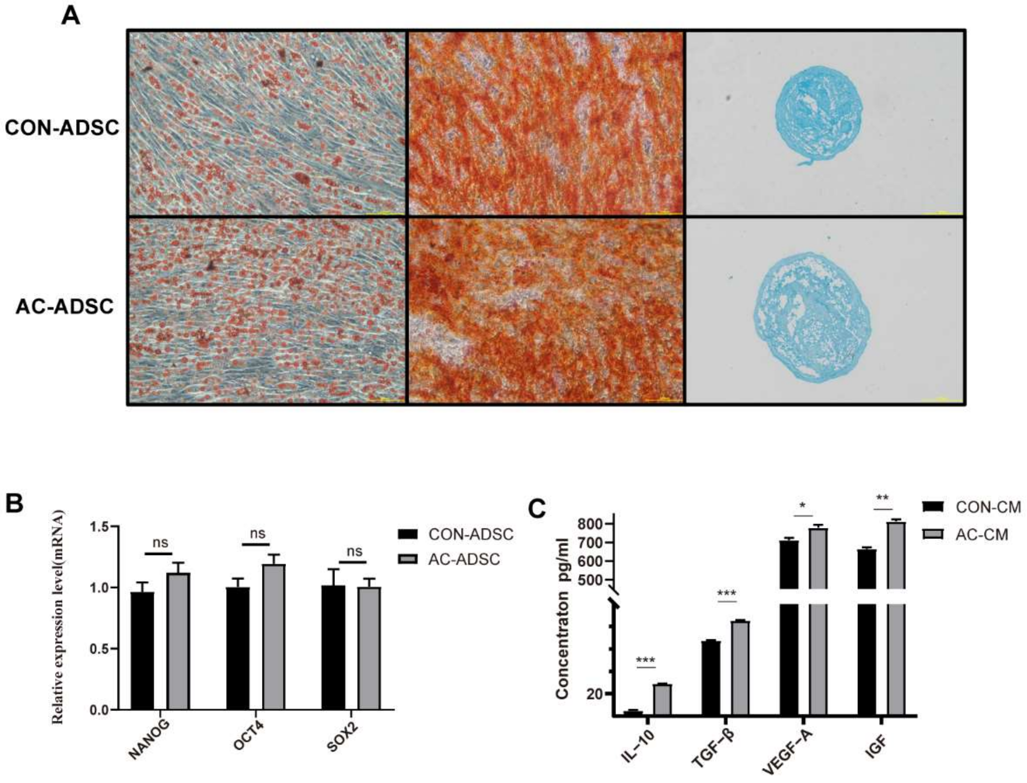

Supplementary fig S1. Stemness maintenance and functional changes of ADSCs after endogenous activation of PDGFR−β. **(A)** Differentiation potential of MSCs in osteogenic, chrondrogenic, and adipogenic lineages using Alizarin red, Alcian blue, and oil red O staining, respectively. **(B)** Expression of *NANOG*, *OCT4*, and *SOX2* detected by RT−qPCR *(n = 3)*. **(C)** Concentration of IL−10, TGF−β, VEGF−A and IGF of conditioned medium detected by ELISA. Data are shown as mean ± SD. Independent-sample t test (two-tailed) was used for statistical comparisons between 2 groups. * $p < 0.05$; ** $p < 0.01$; *** $p < 0.001$.

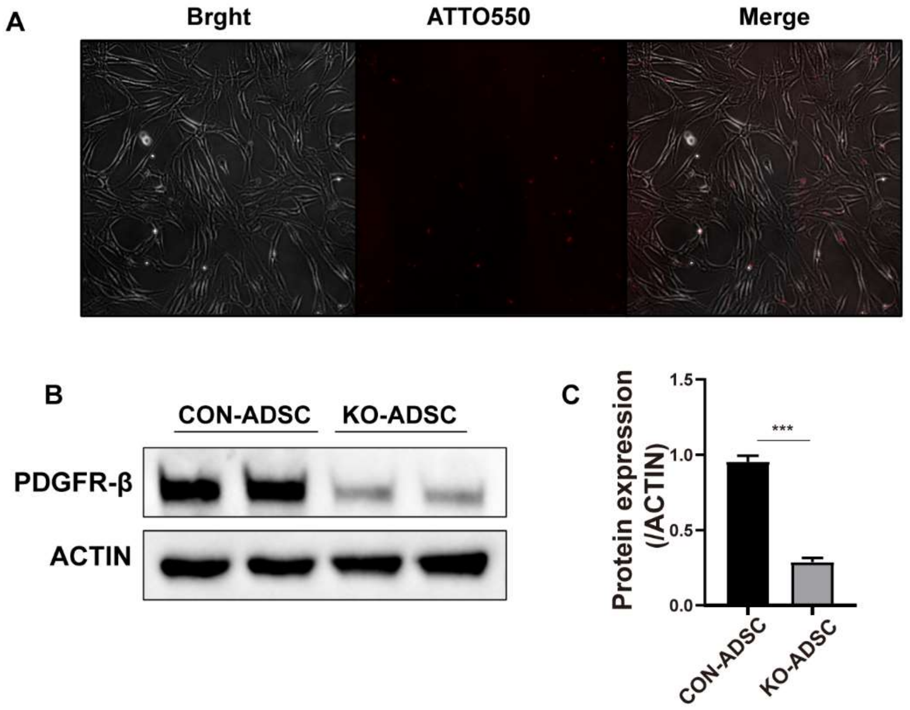

Supplementary fig S2. Knock out of PDGFR-β in ADSCs. **(A)** Representative single-channel and overlay images of ATTO550⁺ ADSCs **(B)** PDGFR-β expression and **(C)** levels relative to ACTIN in ADSCs

Supplementary table S1.

Target gene	Primer sequence
mouse-*col1a1*	CAGAGGCGAAGGCAACA
	GTCCAAGGGAGCCACATC
mouse-*col3a1*	AGAACCTGGCCGAGATG
	TGGACTTCCGGGCATAC
mouse-*tgfb*	TGGGGACTTCTTGGCACT
	ATAGGGGCGTCTGAGGAAC
mouse-*actb*	CCTCACTGTCCACCTTCC
	GGGTGTAAAACGCAGCTC
human-*LN*	CAGACGCACACGGCTCCTAATC
	CCTCGCTGACCTCCTGGATAGTG
human-*VEGFA*	CTGGGCTGTTCTCGCTT
	CCCCTCTCCTCTTCCTTCT
human-*PDGFB*	CACCAACGCCAACTTCC
	GCTTCTTCCGCACAATCTC
human-*ANGPT1*	TGCAATATGGATGTCAATGG
	TATTCACCGGAGGGATTTC
human-*bFGF*	AGAAGAGCGACCCTCACATCA
	CGGTTAGCACACACTCCTTTG
human-*HGF*	GCTATCGGGGTAAAGACCTACA
	CGTAGCGTACCTCTGGATTGC
human-*CXCL12*	ATTCTCAACACTCCAAACTGTGC
	ACTTTAGCTTCGGGTCAATGC
human-*IGF*	GCTCTTCAGTTCGTGTGTGGA
	GCCTCCTTAGATCACAGCTCC

human-*SOX2*	GTGTCAACCTGCATGGC<br>CGAACCATCTCTGTGGTCT
human-*NANOG*	CGGGACCTTGTCTTCCTT<br>CCCTGATTCTTCCACCAGT
human-*OCT4*	CCTGTTCCCCATTCCTAGA<br>CTCCTCAGTCCCTTTCCC
human-*GAPDH*	ACAACTTTGGTATCGTGGAAGG<br>GCCATCACGCCACAGTTTC

Supplementary table S2.

**Antibody**	**Catalog number**	**Company**
PDGFR-β	ET1605-20	HUABIO
PDGFB	ab178409	Abcam
ACTIN	EM21002	HUABIO
GAPDH	ET1601-4	HUABIO
VEGFA	66828-1-Ig	Proteintech
AKT	ET1609-51	HUABIO
p-AKT	ET1607-73	HUABIO
STAT3	#4904	Cell Signaling Technology
p-STAT3	#9145	Cell Signaling Technology
ERK	ET1601-29	HUABIO
p-ERK	ET1610-13	HUABIO
BCL-2	ET1702-53	HUABIO
